# Climate, Toxic Exposures and Work: Linking the Amazon, Africa, and Southern Europe through a COP30 Dialogue

**DOI:** 10.53941/wah.2026.100017

**Published:** 2026-07-31

**Authors:** Roberto G. Lucchini, Sila Apurina, Alexandra Almeida, Silvana Andreescu, Paulo Cesar Basta, Maria Elena Crespo Lopez, Gabriela de Paula Fonseca Arrifano, Alok Deoraj, Enatfenta Sewmehone Endalew, Carlos Espinal, Quentin Felty, Luis Fernandez, Maria Del Carmen Gastañaga, Luca Lombroso, Stefanny Magaly Moncada Barbosa, Ebba Malqvist, Desalew Meseret Moges, Cristina O’Callaghan-Gordo, Jesús Olivero-Verbel, Luciana Pitta, Claudia Vega, Kunihiko Yoshida, Luiz Augusto Galvão, Kurt Straif

**Affiliations:** 1 Department of Environmental Health Sciences, Florida International University, Miami, FL 33199, USA; 2 Occupational Medicine, University of Modena and Reggio Emilia, 41100 Modena, Italy; 3 Amazonian Work Network and Embassy of Indigenous Peoples, Brasilia 70000-000, Brazil; 4 Acción Ecológica, Quito 170129, Ecuador; 5 Chemistry & Biochemistry, Clarkson University, Potsdam, NY 13699, USA; 6 Escola Nacional de Saúde Pública Fundação Oswaldo Cruz, Rio de Janeiro 21041-210, Brazil; 7 Laboratório de Farmacologia Molecular, Amazonian Mercury Institute, Universidade Federal do Pará, Guamá Belém 66077-830, Brazil; 8 School of Nursing, Faculty of Health Sciences, Institute of Health, Jimma University, Jimma P.O. Box 378, Ethiopia; 9 Global Health, Florida International University, Miami, FL 33199, USA; 10 Biology, Sabin Center for Environment and Sustainability, Wake Forest University, Winston-Salem, NC 27109, USA; 11 Centro de Innovación Científica Amazónica (CINCIA), Puerto Maldonado 17001, Peru; 12 Centro Nacional de Salud Ocupacional y Protección del Ambiente para la Salud, Instituto Nacional de Salud, Lima 15073, Peru; 13 ‘Enzo Ferrari’ Engineering, University of Modena and Reggio Emilia, 41100 Modena, Italy; 14 Faculty of Gobierno, Universidad del Desarrollo, Las Condes 7550000, Chile; 15 Occupational and Environmental Medicine, Lund University, 22100 Lund, Sweden; 16 Ethiopian Public Health Institute, Addis Ababa P.O. Box 1242, Ethiopia; 17 Barcelona InTerdisciplinary Research Group on plAnetary heaLth (BITAL), Universitat Oberta de Catalunya, 08018 Barcelona, Spain; 18 Universitat Pompeu Fabra, 08003 Barcelona, Spain; 19 CIBER Epidemiología y Salud Pública (CIBERESP), 28029 Madrid, Spain; 20 Barcelona Institute for Global Health (ISGlobal), 08036 Barcelona, Spain; 21 Environmental and Computational Chemistry Group, Research Institute for Climate Change and Sustainable Development, Universidad de Cartagena. Cartagena 130000, Colombia; 22 Guangwai Law School, Guangdong University of Foreign Studies, Guangzhou 510420, China; 23 Center for International Relations in Health, FIOCRUZ, Rio de Janeiro 21040-900, Brazil

**Keywords:** occupational and environmental health, climate change, occupational heat stress, mercury, microplastics, agrochemicals, Indigenous health

## Abstract

Climate change, toxic pollution, and social inequities are converging to create new risks for workers and communities, particularly in the Global South. Yet occupational and environmental health research and policy still tend to address these stressors in isolation, not considering their interactions. To synthesize the main messages of the COP30 side event, “Amazon and Heat: Community-First Climate–Environment–Health Action from the Amazon Basin to Africa and Europe”, presented in the context of the LIMA2025 Conference on Occupational Medicine, and to propose a framework for integrated climate–environment–health action in work and community settings. This conference report and policy perspective are based on a narrative synthesis of presentations and discussions delivered during the COP30 side event and subsequently presented at LIMA2025 by researchers, Indigenous leaders, occupational health practitioners and civil society organizations from the Amazon Basin, Africa and Europe. Contributions were grouped into thematic domains: (1) climate and toxic exposures in the Amazon; (2) work and heat in African and Southern European settings; and (3) cross-cutting challenges and policy opportunities. The analysis followed a qualitative thematic approach. Across regions, participants described intersecting exposures to heat, toxic chemicals, air pollution, deforestation and psychosocial stress, disproportionately affecting workers, Indigenous peoples, traditional populations, pregnant women and children. Evidence from cohort studies, intervention projects, and remote-sensing analyses highlighted: (i) elevated mercury exposure and increasing microplastics and diesel contamination in Amazonian communities, alongside climate-sensitive food-system vulnerabilities; (ii) growing heat-related health risks among children and pregnant women in African cities; and (iii) dehydration, kidney dysfunction, and cardiometabolic risks among agricultural and construction workers in Southern Europe. Cross-cutting themes included exposure mixtures, inequities in access to care and justice, persistent data gaps, and the need for locally owned technologies, capacity building, and sustainable solutions. The side event catalyzed the development of an Amazon Climate–Environment–Health Consortium and a shared policy agenda. Integrated, equity-focused action on heat, toxic exposures, and work is essential for a just climate transition.

## 1. Introduction

Extreme weather events and climate change are reshaping the risk landscape for workers and communities worldwide. Rising temperatures, more frequent and intense heatwaves, floods, altered precipitation patterns, and extreme weather events interact with existing environmental hazards such as air pollution, toxic chemicals, and land-use change. These converging threats are particularly acute in the Global South, where many workers are employed in outdoor or physically demanding jobs, health systems often operate under resource constraints, and regulatory frameworks may be weak or poorly enforced [1–3].

In this context, the Amazon Basin occupies a central place in planetary health (Figure 1).

The region is a global biodiversity hotspot and a major regulator of the Earth’s climate [4–6], but it is also a frontier of deforestation, informal and industrial mining, oil extraction, agro-industrial expansion, and infrastructure development [7]. These activities have intensified exposures to mercury and other heavy metals, agrochemicals, microplastics, and air pollutants for Indigenous peoples, riverine communities, and workers in the Amazon Basin [8–11]. At the same time, climate change is driving higher temperatures and more frequent extremes, aggravating thermal stress in occupational and community environments [12]. Similar dynamics are emerging in African cities and rural areas, where rapid urbanization, limited green infrastructure and high levels of outdoor work amplify the health impacts of rising heat [13,14]. In Southern Europe, heat waves and changing work organization are contributing to increased risks among agricultural and construction workers, including dehydration and kidney injury [15–18].

Occupational health professionals are uniquely positioned to address these converging challenges because they routinely integrate exposure assessment, prevention strategies, surveillance systems, risk communication, and workplace interventions across diverse environmental and social contexts. To explore these challenges from a multi-regional perspective, a side event entitled “Amazon and Heat: Community-First Climate–Environment–Health Action from the Amazon Basin to Africa and Europe” was held at the 30th Conference of the Parties (COP30) in Belém, Brazil, on 19 November 2025. The event brought together researchers, Indigenous leaders, occupational physicians, public health practitioners and civil society organizations from Brazil and other Amazonian countries, several African nations and European institutions.

At the LIMA2025 Conference held by the Scientific Committee on Occupational Medicine (SCOM) of the International Commission on Occupational Health (ICOH) in Lima, Peru, on 27–29 November 2025, we aimed to: (1) synthesize the key scientific and policy messages that emerged from the COP30 side event, focusing on the nexus of climate change, toxic exposures and work; (2) highlight common challenges and opportunities across the Amazon, Africa and Europe; and (3) present the rationale for an Amazon Climate–Environment–Health Consortium, in dialogue with African and European partners. A detailed policy brief that translates these insights into concrete recommendations for decision-makers is provided as a Supplementary Document. This manuscript should therefore be read as a conference-derived narrative synthesis and policy perspective, not as original human-subjects research or a systematic review.

## 2. Materials and Methods

This article is based on a narrative synthesis of a 90-min side event held during COP30 of the UN Framework Convention on Climate Change, at the Italian Ministry of Environment’s pavilion in Belém, Brazil. Speakers included academic researchers, Indigenous leaders, public health practitioners, and civil society representatives from the Amazon Basin, Africa, and Europe. The manuscript does not report original data collection or a formal systematic review. Instead, it synthesizes evidence, expert perspectives, and policy priorities presented during the side event. Published studies are cited where empirical evidence is discussed, whereas conference-derived observations and recommendations are identified as such. Presentations covered empirical studies, intervention projects, innovations in sensing technologies for real-time monitoring of toxic pollutants, and community-based assessments. Contributions were grouped thematically into three thematic domains rather than formally coded: (1) climate and toxic exposures in the Amazon, (2) work and heat in Africa and Southern Europe, and (3) cross-cutting themes and policy implications. Given the diversity of designs and the descriptive nature of most contributions, we used a qualitative thematic synthesis approach. All presentations delivered during the COP30 side event were considered for inclusion in the synthesis, which was developed through discussion among the authors and refined during manuscript preparation. Because the objective was to synthesize key scientific and policy messages rather than conduct a formal qualitative research study, no structured coding framework or inter-rater reliability assessment was applied. Differences in interpretation were resolved through discussion and consensus among co-authors. We acknowledge that conference-based syntheses may be affected by selection bias, as participants represent invited speakers and attendees and therefore may not capture the full diversity of perspectives across the Amazon Basin, Africa, and Europe. A detailed policy brief is provided as a Supplementary Document.

No individual-level identifiable data are presented, and all empirical studies referenced had received local ethical approval.

## 3. Results

The results below summarize the main thematic messages emerging from the side event. They combine evidence from published studies, ongoing projects, and speaker presentations; policy proposals are identified as recommendations or future directions rather than empirical findings.

### 3.1. Climate, Toxic Exposures, and Equity in the Amazon Basin

#### 3.1.1. Multiple Hazards: Heat, Mercury, and Co-Exposures

The speakers from Brazil, Colombia, Peru and Ecuador described a multi-hazard context in which: (i) informal and industrial gold mining release large quantities of mercury into air, soil and water; (ii) oil extraction, agro-industrial expansion and deforestation introduce additional chemical, physical and mental stressors; (iii) climate change increases ambient temperatures and alters hydrological cycles, affecting the behaviour and bioavailability of contaminants.

Biomonitoring data from Indigenous and riverine communities in the Amazon indicate persistently elevated mercury exposure, especially among populations whose diet depends heavily on fish [8,19–22]. As shown in Table 1, average hair mercury concentrations reported for Amazonian populations reach 15.0 ppm (equivalent to 15.0 μg/g) in Brazil, 11.83 ppm in Peru, and 9.13 ppm in Colombia, exceeding commonly cited health-based guidance values such as weekly tolerable intake recommendations of U.S. EPA and FAO/WHO, equivalent respectively to 1.0 and 2.3 ppm of hair mercury [23], and surpassing recent averages reported in Japan, the Seychelles, the Faroe Islands, and Italy [24–27]. Although mercury levels in hair have declined in these previously highly exposed populations following the application of dietary policies, they remain much higher in the Amazonian populations. Throughout the manuscript, ppm and μg/g are numerically equivalent for hair mercury measurements.

Conversely, fish mercury concentrations remain relevant and unchanged globally, and often quite high, especially in large predatory species [28]. Although direct longitudinal analyses linking annual gold prices to fish mercury concentrations in the Amazon remain limited, available evidence supports a plausible economic-environmental pathway in which higher gold prices stimulate industrial and informal gold mining and, consequently, mercury use and release. In the Peruvian Amazon, rising international gold prices were strongly linked to mining expansion and to national mercury imports used for artisanal mining; Swenson et al. (2011) reported that gold price was associated with exponential increases in Peru’s mercury imports from 2003 to 2009 (R^2^ = 0.93, *p* = 0.04) [29]. Mercury used for amalgamation is dispersed to air [30], soil [31], and aquatic systems, where it can be transformed and bioaccumulate in fish. Therefore, increasing gold prices should be considered a likely upstream driver of higher fish mercury contamination risk in mining-affected Amazonian areas, even if the fish response is shaped by species, trophic level, hydrology, and local biogeochemical conditions.

The particularly high burden in the Amazon is consistent with the mercury content of commonly consumed large predatory freshwater fish, such as Dourada and Filhote/Piraíba, whose average concentrations commonly range from about 1.8 to 3.6 mg/kg, while Babão averages around 2.9 mg/kg [20]. Important differences persist globally by species and market type, with fresh large predators generally showing substantially higher mercury concentrations than canned fish, while canned tuna tends to have lower average levels overall, although Albacore (‘white’) tuna remains higher than light/skipjack tuna products [32,33].

Portable and smartphone-enabled biosensors were discussed as tools for rapid on-site screening of mercury, PFAS and other contaminants in remote settings [42–44]. This technology will allow sustainable assessment by trained local scientists, yielding further knowledge of chemical risks from various environmental media and biomarkers.

In parallel, climate projections and observational evidence indicate increasing temperatures, higher heat stress indices, and more frequent extreme heat events across parts of the Amazon Basin [45]. Long-term meteorological observations from northern Italy were used to illustrate how regional warming can be interpreted within broader climate, ecosystem, and health processes [46]. Integrating meteorological monitoring, climatology, and ecosystem perspectives is key to understanding how regional warming connects with global environmental change and its health implications. The discussion emphasized the interaction between mercury contamination and climate change in the Amazon, where gold mining remains a major source of mercury release while climate-driven changes in temperature, hydrology, and forest dynamics may alter mercury cycling, remobilization, and bioavailability. Forests can function both as a sink and a secondary source of mercury, raising concern that ongoing environmental change may amplify human exposure in already vulnerable communities. Therefore, an integrated response is highly needed, combining health assessment and human biomonitoring programs to prevent intoxication, awareness materials for affected populations, and legislative initiatives to strengthen prevention and control. This integrated approach is currently conducted at the Amazonian Mercury Institute (Instituto Amazónico do Mercurio, IAMER) [47].

The 2023 Amazon crisis, marked by record heat and drought, illustrates how these hazards are already emerging in routine exposure rather than remaining only in future scenarios [45]. For outdoor workers such as miners, loggers, and agricultural laborers, higher ambient heat and humidity increase the risks of heat strain, dehydration, impaired work capacity, and occupational injury [16,17]. Households without adequate housing quality or access to cooling are also more vulnerable, as extreme heat is intensified by poor thermal protection and limited adaptive capacity [48,49].

Panelists stressed that besides mercury, communities are concurrently exposed to other chemicals, noise and psychosocial stress, creating complex mixtures whose combined effects are still poorly understood. Pesticide contamination has been documented in water and sediments on Indigenous lands in the Brazilian Amazon, and recent biomarker-based studies in traditional Amazonian communities suggest that environmental contamination can be accompanied by measurable effect biomarkers, including altered cholinesterase activity and related inflammatory markers [50]. Microplastics are now recognized as widespread contaminants across Amazonian aquatic and terrestrial ecosystems [11]. Recent evidence reveals the presence of ‘forever chemicals’ (e.g., perfluoroalkyl and polyfluoroalkyl substances, PFAS) in the Amazon Forest, with detectable levels of perfluorooctanoic acid (PFOA) at concentrations up to 2 pg/m^3^ [51]. However, the impacts of PFAS exposure in this ecosystem remain largely unknown.

Seasonal biomass burning further increases particulate air pollution and is associated with adverse respiratory and cardiovascular effects, with disproportionate impacts on Indigenous populations and other highly exposed communities [52–54]. In addition, environmental degradation, extractive activities, and repeated contamination events can generate chronic psychosocial stress and mental health burdens, underscoring the need to address these exposures within a broader climate–environment–health exposome [53]. Decades of oil extraction in the Ecuadorian Amazon have contaminated water, soil, and air, degraded biodiversity, and contributed to stress, anxiety, depression, and feelings of powerlessness, particularly among women in affected Indigenous communities. These findings suggest that environmental degradation should be considered not only a source of toxic exposures but also a determinant of mental health, acting through chronic stress pathways that interact with social vulnerability, displacement, loss of livelihoods, and cultural disruption.

#### 3.1.2. Indigenous and Traditional Populations’ Health, Rights and Environmental Justice

Emerging evidence links these exposures to neurodevelopmental effects, as well as possible cardiovascular and metabolic outcomes [8,21,55,56]. Evidence from fieldwork in Indigenous territories, particularly among the Munduruku population, reveals: (i) high levels of in utero and early-life mercury exposure, with clinical pictures reminiscent of congenital Minamata disease in some children; (ii) encroachment of illegal mining into protected areas and Indigenous lands; and (iii) inadequate access to basic services such as safe drinking water, fresh food and transportation [19,20,55]. Preliminary results from a longitudinal study of Indigenous pregnant women and newborns further underscore the magnitude of this burden: among 178 pregnant women, hair total mercury showed a median of 8.88 μg/g, an average of 9.89 μg/g, and a maximum of 31.0 μg/g, while among their 125 newborns the median was 5.52 μg/g, the average 5.83 μg/g, and the maximum 16.1 μg/g, all well above the weekly tolerable intake recommendations. These findings support concern that mercury exposure begins during gestation and continues through the first 1000 days of life, with potentially serious implications for neurodevelopment and child health in communities already affected by structural barriers to healthcare access [55].

Additional evidence from the Colombian Amazon and other mining-affected Amazonian regions shows that mercury exposure extends far beyond a conventional toxicological problem; it represents a climate-sensitive, food-system-mediated public health threat that disproportionately burdens Indigenous and riverine communities, particularly pregnant women, infants, and children [19,55,57]. Community-based biomonitoring has repeatedly documented hair mercury concentrations above health-based benchmarks in populations that depend heavily on fish as a primary source of animal protein, while territorial isolation, limited access to safer dietary alternatives, and hydrological instability associated with climate variability further deepen vulnerability [8,19,20,22,49,55,57]. This pattern calls for integrated surveillance frameworks linking human biomonitoring, watershed-based exposure assessment, culturally grounded risk communication, and locally adapted dietary guidance, with particular emphasis on protection during pregnancy and the first 1000 days of life [8,55,57]. Taken together, these conditions position the Amazon as a critical setting where environmental toxicology, climate variability, food security, and environmental justice converge within the same public health and policy challenge.

From a legal perspective, participants noted that the local judicial mechanisms have often been slow, difficult to access, and insufficient to secure timely remediation and compensation for Indigenous and riverine communities affected by extractive activities in the Amazon [58,59]. Inter-American human rights reports on the Pan-Amazon have called for decisive action against impunity and for both individual and collective redress for victims of business-related and illegal activities, while the UN Office of the High Commissioner for Human Rights (OHCHR) guidance emphasizes that access to remedy should include effective non-judicial mechanisms in addition to courts [60]. In this context, panelists argued for culturally appropriate administrative compensation registries or claims mechanisms specifically tailored to mercury poisoning linked to mining and oil extraction, combined with immediate investments in safe water, food access, transport, and health services.

#### 3.1.3. Transport Corridors, Diesel Pollution and “Logistics Heat Islands”

An Indigenous-led assessment showed that major federal highways in the Brazilian Amazon (BR-319, BR-174, BR-163, and BR-010/316/222) function as intensive diesel truck corridors serving export-oriented supply chains. Estimates suggest that nearly 12,000 heavy diesel trucks operate along the four major transport corridors, generating approximately 1.5 million tons of CO_2_ per year [61]. Beyond greenhouse gas emissions, they create air pollution hotspots (fine particles, nitrogen oxides), noise, soil and water contamination from fuel and lubricants, and “logistics heat islands” around industrial districts and logistic hubs. Health impact calculations indicate substantial burdens of respiratory morbidity and mortality in populations living near these corridors. The analysis also showed that fiscal incentives and tax exemptions subsidize this high-carbon logistics model, while externalizing health and environmental costs to the local communities [61].

### 3.2. Africa and Europe: Heat, Work and Vulnerable Populations

#### 3.2.1. Child Heat Impacts and School-Based Interventions in Africa

The Child Heat Impacts—Climate Health Interventions (CHI^2^) programme [62] led by the University of KwaZulu-Natal highlights children’s vulnerability to rising temperatures in African settings, where school environments, limited shade, classroom design, and urban heat can intensify heat-related symptoms and respiratory complaints. Its school-based and community-engaged approach emphasizes shared themes of heat vulnerability, urban design, and locally co-designed interventions, including shaded spaces, cooling strategies, adapted timetables, and child-friendly heat awareness tools.

#### 3.2.2. Urban Heat Islands and Green Infrastructure in Ethiopian Cities

Remote-sensing analyses the ENABLE project in four Ethiopian cities highlighted how rapid urbanization and uneven green infrastructure shape urban heat vulnerability, with higher surface temperatures in peripheral expanding areas and daytime cooling associated with vegetation expansion [63,64]. These findings reinforce the importance of urban design, green corridors, and spatial planning as practical interventions to reduce heat exposure and related non-communicable disease risks in rapidly growing cities.

#### 3.2.3. Pregnant Women and Agricultural Workers as Sentinel Groups

Heat-focused studies in urban Ethiopia and rural Southern Spain identified pregnant women and female agricultural workers as sentinel groups for climate-related health risks, linking urban heat islands, limited cooling, poor walkability, and high physical workloads to constrained mobility, dehydration, heat strain, and early kidney dysfunction. Together, these findings emphasize shared needs for heat-sensitive urban design, safer work organization, hydration and pacing strategies, and stronger intervention evidence for vulnerable workers and populations [65].

#### 3.2.4. Occupational Heat Strain in Construction and Other Outdoor Work

The INTERCAMBIO living review on heat-stress interventions for construction workers highlights a major intervention gap: few studies exist, most focus on personal protective equipment, and evidence remains limited for multidimensional approaches, low- and middle-income countries, and women workers [66]. This reinforces the need for co-created heat-prevention plans that combine engineering controls, safer work organization, hydration and pacing strategies, and regulatory measures, an area where coordinated studies through the proposed Consortium could add value.

### 3.3. Cross-Cutting Themes

Across all regions and presentations, several common themes emerged.

Mixture exposomic effects: Populations are rarely exposed to a single hazard. Heat interacts with chemicals, air pollution, heavy workloads and psychosocial stress, yet risk assessments and regulations still largely focus on single agents. Emerging evidence indicates the presence of novel contaminants, including microplastics and “forever chemicals”. However, their interactions with legacy pollutants such as mercury are largely unknown, representing a critical knowledge gap with potentially significant consequences for ecosystem and human health.

Inequities in exposure, care and justice: Indigenous peoples, low-income workers, informal laborers, women and children bear the brunt of converging hazards, while benefiting least from economic activities that drive them. Access to health care, legal remedies and compensation is uneven and often insufficient.

Data gaps and fragmented evidence: There is a shortage of robust, longitudinal and intervention studies on heat and mixed exposures in low- and middle-income countries, and a lack of standard indicators for children’s heat-related health and community-level impacts.

Technology and local ownership: Wearables, remote sensing, biosensors and omics platforms can support “precision public health”, but only if embedded in locally owned systems and accompanied by capacity building and open, equitable data governance. Portable sensors can be especially powerful for community-based monitoring by overcoming the limitations of traditional laboratory-based techniques and enabling cost-effective measurements in remote areas [67,68].

Transdisciplinary and trans-regional collaboration: The complexity of climate–environment–health challenges require collaboration across disciplines (epidemiology, toxicology, climate science, social science, law) integrated with Indigenous knowledge and across regions.

## 4. Discussion

The COP30 side event illustrated that the interface between climate, toxic exposures and work is increasingly central to global occupational and environmental health yet remains under-addressed in both research and policy. The Amazon Basin exemplifies how climate change, extractive economies and infrastructural development converge to produce multi-hazard environments. Air pollution and water contamination from the oil industry, mercury exposure, diesel emissions, deforestation, and rising temperatures jointly threaten the health of workers and communities, with impacts on Indigenous people and traditional communities. Emerging pollutants and their impacts on these ecosystems pose an additional and growing challenge. These risks are especially critical where fish-dependent food systems, maternal-child vulnerability, and limited access to health services converge, reinforcing the need for integrated biomonitoring and culturally grounded prevention strategies. In African and European settings, heat-related risks in construction, agriculture, and urban environments demonstrate that even in diverse socio-economic contexts, workers and vulnerable populations face common threats. While the African examples presented here focused primarily on heat-related health risks and urban adaptation, relatively little evidence is currently available on combined climate and toxic chemical exposures in African populations, highlighting an important research gap. Ethiopia, for example, is highly exposed to toxic substances from air pollution, which was not covered in this meeting due to time limitations.

The occupational health community is well positioned to contribute frameworks and tools—such as exposure assessment methods, hazard control hierarchies, and surveillance systems—that can be adapted to this broader climate–environment–health agenda. However, these tools must be expanded to account for mixtures of exposures and structural determinants, including legal, economic, and political intergovernmental relations.

An exposomic perspective—capturing the totality of environmental exposures across the life course—provides a unifying framework to characterize these complex interacting risks. Applying exposomics can strengthen the assessment of cumulative and co-occurring hazards (e.g., heat, air pollution, and chemical exposures) and link them more directly to health outcomes, thereby informing more integrated prevention and policy strategies. For example, integrating biomonitoring of mercury, pesticides, PFAS, and inflammatory biomarkers with wearable heat sensors, satellite-derived environmental indicators, and multi-omics approaches could help characterize cumulative exposures and identify vulnerable populations across the life course.

This conference-based synthesis has limitations. It reflects the topics and perspectives represented at the side event and is not a systematic review of all available evidence. Some regions and hazards are therefore discussed in greater depth than others, particularly the Amazonian mercury and extractive-sector issues that were central to the event.

A key proposal emerging from the side event was the establishment of an Amazon Climate–Environment–Health Consortium as an organizing platform aimed at achieving several goals:

- Connect existing and emerging cohorts and community-based studies across the Amazon Basin;
- Harmonize existing data, environmental and biomonitoring protocols for mercury, other contaminants, air pollution, and heat;
- Develop an integrated framework for community-based monitoring and assessment of ecosystem health.
- Integrate Indigenous knowledge and social science perspectives;
- Respect local expertise while strengthening local infrastructure and supporting the retention of qualified human resources;
- Coordinate training and capacity-building activities;
- Co-produce policy-relevant evidence with African and European partners facing analogous issues.

If implemented, such a consortium could serve as a bridge between research and policy, by generating the evidence base required to support the recommendations summarized in the accompanying policy brief (Supplementary Document). These include stronger control of mercury and other chemicals in mining and oil extraction, identification of emerging hazards, just transitions in transport and logistics, enhanced occupational heat protections, and shared indicators and data infrastructures.

## 5. Conclusions

This conference report and policy perspective synthesizes discussions from the COP30 side event on “Amazon and Heat”, which brought together diverse stakeholders from the Amazon Basin, Africa, and Europe to examine how climate change, toxic exposures, and working conditions intersect. The discussions highlighted that heat is a unifying risk factor across regions and sectors, but its impacts are magnified where workers and communities are also exposed to toxic chemicals, air pollution and social vulnerability. Framing these intersecting risks through an exposomic lens can enhance understanding of cumulative, life-course exposures and their combined health effects, enabling more precise identification of vulnerable populations and more integrated intervention strategies across climate and occupational/environmental health domains. The proposed Amazon Climate–Environment–Health Consortium aims to operationalize these insights by linking epidemiological, environmental, and social science work across multiple countries, while the policy brief (Supplementary Document) translates them into actionable recommendations. Protecting Amazonian forests, African urban communities and workers on all continents from the combined risks of heat and pollution is both a public health imperative and a precondition for a just climate transition.

## Supplementary Material

Supplementary Material

The additional data and information can be downloaded at: https://media.sciltp.com/articles/others/2607310939374747/WAH-26050040-SM.pdf. Policy Brief—“Amazon and Heat: Community-First Climate–Environment–Health Action from the Amazon Basin to Africa and Europe”.

## Figures and Tables

**Figure 1.**
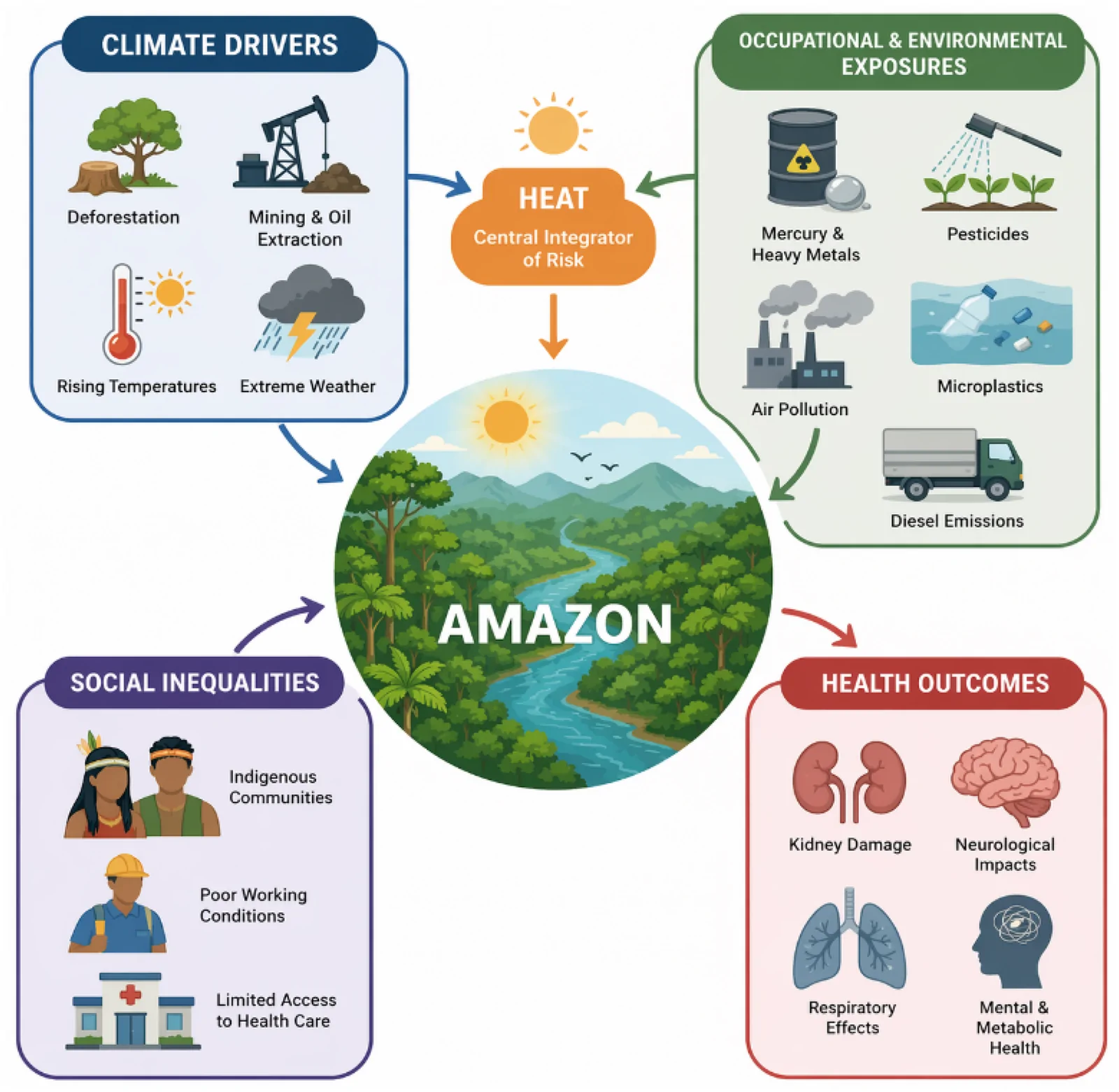
Conceptual framework illustrating interactions among climate drivers, occupational and environmental exposures, social inequalities, and health outcomes in the Amazon Basin. Climate change, deforestation, mining, and industrial activities contribute to multiple exposures—including mercury, pesticides, PFAS, air pollution, microplastics, and heat—which disproportionately affect vulnerable populations and contribute to neurological, respiratory, metabolic, renal, and mental health outcomes.

**Table 1.** Comparative average mercury levels in human hair across selected populations. Values reported for Amazonian populations substantially exceed commonly cited health-based benchmarks of 1.0 ppm (U.S. EPA equivalent) and 2.3 ppm (FAO/WHO equivalent).

Population/Region	Average Hair Hg (ppm)	Context	References
Brazil	15.0	Amazonian populations	[34–37]
Peru	11.8	Amazonian populations	[38]
Colombia	9.1	Amazonian populations	[8]
Japan	2.01 men; 1.85 women	Current levels after major decline from Minamata values of ~30 ppm	[24]
Seychelles	2.5	After reduced fish intake (down from ~7 ppm)	[25]
Faroe Islands	~1.00	After reduced pilot whale intake (down from ~5 ppm)	[26,39]
Italy	1.65 (>4 monthly fish meals); 1.40 (<4)	General population study	[27]
Guidance value	1.0	Equivalent to U.S. EPA tolerable intake recommendation	[23,40]
Guidance value	2.3	Equivalent to FAO/WHO tolerable intake recommendation	[23,41]

## References

[1] Ioannou LG; Foster J; Morris NB; Occupational heat strain in outdoor workers: A comprehensive review and meta-analysis. Temperature 2022, 9, 67–102. 10.1080/23328940.2022.2030634. PMC9154804 35655665

[2] Myhre S; Frønsdal K; Ames H; Building and Strengthening Climate-Resilient Health Systems in Low- and Middle-Income Country Contexts; Norwegian Institute of Public Health: Oslo, Norway, 2024.

[3] Gray WB; Shadbegian R. Economics of environmental compliance and enforcement. In Oxford Research Encyclopedia of Environmental Science; Oxford University Press: Oxford, UK, 2021.

[4] Flores BM; Montoya E; Sakschewski B; Critical transitions in the Amazon forest system. Nature 2024, 626, 555–564. 10.1038/s41586-023-06970-0. PMC10866695 38356065

[5] Artaxo P; Hansson HC; Machado LAT; Amazon deforestation implications for local and regional climate: A review. Theor. Appl. Climatol. 2023, 153, 317–333.

[6] Hacón S; Rivera GA; Rodrigues YE; Health in the Amazon: Environmental, Social and Climate Determinants; Science Panel for the Amazon: New York, NY, USA, 2024.

[7] Bebbington AJ; Verdum R; Gamboa C; Resource extraction and infrastructure threaten forest cover and community rights. Proc. Natl. Acad. Sci. USA 2018, 115, 13164–13173. 10.1073/pnas.1812505115. PMC6310830 30509982

[8] Lucchini RG; Basta PC; Crespo-Lopez ME; Mercury Contamination and Co-exposures in the Amazon Basin: At the Center of the Planetary Environmental Crisis. Ann. Glob. Health 2025, 91, 42. 10.5334/aogh.4817. PMC12315690 40756479

[9] Moulatlet GM; Yacelga N; Rico A; A systematic review on metal contamination due to mining activities in the Amazon basin and associated environmental hazards. Chemosphere 2023, 339, 139700. 10.1016/j.chemosphere.2023.139700. 37532203

[10] Damiani S; do Nascimento RF; de Souza CMM; Water and sediment pesticide contamination on Indigenous lands in the Brazilian Amazon. Heliyon 2023, 9, e21445. 10.1016/j.heliyon.2023.e21445. PMC10522942 37771527

[11] Morais LMS; Queiroz A; Brito BKF; Microplastics in the Amazon biome: State of the art and future priorities. Heliyon 2024, 10, e28851. 10.1016/j.heliyon.2024.e28851. PMC11002258 38596029

[12] Cissé G; McLeman R; Adams H; Health, Wellbeing and the Changing Structure of Communities. In Climate Change 2022: Impacts, Adaptation and Vulnerability; Chapter 7; Cambridge University Press: Cambridge, UK; New York, NY, USA, 2022.

[13] Li X; Stringer LC; Dallimer M; The role of blue-green infrastructure in the urban thermal environment across African cities. Sustain. Cities Soc. 2022, 80, 103780. 10.1016/j.scs.2022.103780.

[14] Habibi P; Moradi G; Dehghan A; The impacts of climate change on occupational heat strain in outdoor workers: A systematic review. Urban Clim. 2021, 39, 100928.

[15] Climate-ADAPT. Effects on Occupational Health and Safety; Climate-ADAPT: Copenhagen, Denmark, 2023.

[16] Gibb K; Beckman S; Vergara XP; Extreme heat and occupational health risks. Annu. Rev. Public Health 2024, 45, 59–78. 10.1146/annurev-publhealth-060222-034715 38166501

[17] Nerbass FB; Pecoits-Filho R; Clark WF; Occupational heat stress and kidney health: From farms to factories. Kidney Int. Rep. 2017, 2, 998–1008. 10.1016/j.ekir.2017.08.012. PMC5733743 29270511

[18] Roca J; Sanromà-Ortiz M; Cemeli T; Health interventions for the prevention of dehydration in agricultural workers exposed to heat stress: A systematic review. Healthcare 2025, 13, 1137. 10.3390/healthcare13111232 PMC12155329 40508846

[19] Basta PC; Viana PVS; Vasconcellos ACS; Mercury Exposure in Munduruku Indigenous Communities from Brazilian Amazon: Methodological Background and an Overview of the Principal Results. Int. J. Environ. Res. Public Health 2021, 18, 9222. 10.3390/ijerph18179222. PMC8430525 34501811

[20] de Vasconcellos ACS; Hallwass G; Bezerra JG; Health Risk Assessment of Mercury Exposure from Fish Consumption in Munduruku Indigenous Communities in the Brazilian Amazon. Int. J. Environ. Res. Public Health 2021, 18, 7940. 10.3390/ijerph18157940 PMC8345402 34360233

[21] Lopes-Araújo A; Arrifano GP; Macchi BM; Hair mercury is associated with dyslipidemia and cardiovascular risk: An anthropometric, biochemical and genetic cross-sectional study of Amazonian vulnerable populations. Environ. Res. 2023, 229, 115971. 10.1016/j.envres.2023.115971. 37105291

[22] Santos-Sacramento L; Arrifano GP; Lopes-Araújo A; Human neurotoxicity of mercury in the Amazon: A scoping review with insights and critical considerations. Ecotoxicol. Environ. Saf. 2021, 208, 111686. 10.1016/j.ecoenv.2020.111686. 33396018

[23] Crespo-Lopez ME; Augusto-Oliveira M; Lopes-Araújo A; Mercury: What can we learn from the Amazon? Environ. Int. 2021, 146, 106223. 10.1016/j.envint.2020.106223. 33120229

[24] Takeuchi H; Shiota Y; Yaoi K; Mercury levels in hair are associated with reduced neurobehavioral performance and altered brain structures in young adults. Commun. Biol. 2022, 5, 529. 10.1038/s42003-022-03464-z. PMC9163068 35655003

[25] Klus JK; Thurston SW; Myers GJ; Postnatal methylmercury exposure and neurodevelopmental outcomes at 7 years of age in the Seychelles Child Development Study Nutrition Cohort 2. Neurotoxicology 2023, 99, 115–119. 10.1016/j.neuro.2023.10.004. PMC10842381 37832849

[26] Weihe P; Joensen HD. Dietary recommendations regarding pilot whale meat and blubber in the Faroe Islands. Int. J. Circumpolar Health 2012, 71, 18594. 10.3402/ijch.v71i0.18594. PMC3417701 22789518

[27] Cegolon L; Petranich E; Pavoni E; Concentration of mercury in human hair and associated factors in residents of the Gulf of Trieste (North-Eastern Italy). Environ. Sci. Pollut. Res. Int. 2023, 30, 21425–21437. 10.1007/s11356-022-23384-z. PMC9938062 36269483

[28] Basta PC; de Vasconcellos ACS; Hallwass G; Risk Assessment of Mercury-Contaminated Fish Consumption in the Brazilian Amazon: An Ecological Study. Toxics 2023, 11, 800. 10.3390/toxics11090800. PMC10535031 37755810

[29] Swenson JJ; Carter CE; Domec JC; Gold mining in the Peruvian Amazon: Global prices, deforestation, and mercury imports. PLoS ONE 2011, 6, e18875. 10.1371/journal.pone.0018875. PMC3079740 21526143

[30] Szponar N; Vega CM; Gerson J; Tracing Atmospheric Mercury from Artisanal and Small-Scale Gold Mining. Environ. Sci. Technol. 2025, 59, 5021–5033. 10.1021/acs.est.4c10521. 40043167

[31] Rodriguez-Pascual MJ; Vega C; Andrade N; Effects of mercury exposure in three Amazonian agricultural plant species and the impact on bacterial population in soil. Environ. Res. 2025, 285, 122469. 10.1016/j.envres.2025.122469. 40738411

[32] FDA. Mercury Levels in Commercial Fish and Shellfish (1990–2012); U.S. Food and Drug Administration: Silver Spring, MD, USA, 2022.

[33] FDA; EPA. Questions and Answers from the FDA/EPA Advice about Eating Fish for Those Who Might Become or Are Pregnant or Breastfeeding and Children Ages 1 to 11 Years; U.S. Food and Drug Administration: Silver Spring, MD, USA; U.S. Environmental Protection Agency: Washington, DC, USA, 2022.

[34] Vega CM; Orellana JDY; Oliveira MW; Human Mercury Exposure in Yanomami Indigenous Villages from the Brazilian Amazon. Int. J. Environ. Res. Public Health 2018, 15, 1051. 10.3390/ijerph15061051. PMC6028914 29789499

[35] da Conceição Nascimento Pinheiro M; Cardoso FC; do Nascimento de Aviz LB; Mercury Exposure and Health Effects in Indigenous People from the Brazilian Amazon-Literature-Scoping Review. Int. J. Environ. Res. Public Health 2025, 22, 1159. 10.3390/ijerph22081159. PMC12386174 40869747

[36] Rebouças BH; Kubota GT; Oliveira RAA; Long-Term Environmental Methylmercury Exposure Is Associated with Peripheral Neuropathy and Cognitive Impairment among an Amazon Indigenous Population. Toxics 2024, 12, 212. 10.3390/toxics12030212. PMC11154458 38535945

[37] Jacques AD; Furutani de Oliveira MA; da Silva MC; Clinical, Laboratory and Neurodevelopmental Findings in Children from the Yanomami-Ninam Population Chronically Exposed to Methylmercury. Toxics 2024, 12, 193. 10.3390/toxics12030193. PMC10975939 38535926

[38] Silman AK; Chhabria R; Hafzalla GW; Impairment in Working Memory and Executive Function Associated with Mercury Exposure in Indigenous Populations in Upper Amazonian Peru. Int. J. Environ. Res. Public Health 2022, 19, 10989. 10.3390/ijerph191710989. PMC9517927 36078698

[39] Weihe P; Grandjean P; Jørgensen PJ. Application of hair-mercury analysis to determine the impact of a seafood advisory. Environ. Res. 2005, 97, 200–207. 10.1016/j.envres.2004.01.006. 15533336

[40] National Research Council (US) Committee on the Toxicological Effects of Methylmercury. Toxicological Effects of Methylmercury; National Academies Press (US): Washington, DC, USA, 2000. 25077280

[41] FAO; WHO. Evaluation of Certain Food Additives and Contaminants: Sixty-First Report of the Joint FAO/WHO Expert Committee on Food Additives; FAO: Rome, Italy; WHO: Geneva, Switzerland, 2003

[42] Wei Q; Nagi R; Sadeghi K; Detection and Spatial Mapping of Mercury Contamination in Water Samples Using a Smart-Phone Based Sensor. ACS Nano 2014, 8, 1121–1129. 10.1021/nn406571t. PMC3949663 24437470

[43] Ateia M; Wei H; Andreescu S. Sensors for Emerging Water Contaminants: Overcoming Roadblocks to Innovation. Environ. Sci. Technol. 2024, 58, 2636–2651. 10.1021/acs.est.3c09889. 38302436

[44] Sarwar M; Leichner J; Naja GM; Smart-phone, paper-based fluorescent sensor for ultra-low inorganic phosphate detection in environmental samples. Microsyst. Nanoeng. 2019, 5, 56. 10.1038/s41378-019-0096-8. PMC6803704 31645999

[45] Espinoza JC; Jimenez JC; Marengo JA; The new record of drought and warmth in the Amazon in 2023 related to regional and global climatic features. Sci. Rep. 2024, 14, 11203. 10.1038/s41598-024-58782-5. PMC10998876 38582778

[46] Costanzini S; Boccolari M; Vega Parra S; A comparative analysis of temperature trends at Modena Geophysical Observatory and Mount Cimone Observatory, Italy. Int. J. Climatol. 2024, 44, 4741–4766. 10.1002/joc.8607.

[47] Instituto Amazônico do Mercúrio (IAMER). Available online: https://www.instagram.com/iamer.amazon/ (accessed on 1 July 2026).

[48] Sustainable Energy for All. Chilling Prospects 2023: Access to Cooling Gaps and Opportunities; Sustainable Energy for All: Vienna, Austria, 2023.

[49] Augusto-Oliveira M; Arrifano G; Leal-Nazaré C; Human methylmercury exposure/intoxication in the Global South: Insights for prevention/mitigation in the Amazonian context. Sci. Total Environ. 2025, 1001, 180537. 10.1016/j.scitotenv.2025.180537. 40992186

[50] Fona CMT; Miranda AMM; Jesus MI; Biomarkers of Pesticide Exposure in a Traditional Brazilian Amazon Community. Int. J. Environ. Res. Public Health 2024, 21, 1396. 10.3390/ijerph21111396. PMC11593667 39595663

[51] Kourtchev I; Sebben BG; Brill S; Occurrence of a “forever chemical” in the atmosphere above pristine Amazon Forest. Sci. Total Environ. 2024, 944, 173918. 10.1016/j.scitotenv.2024.173918. 38866151

[52] Urrutia-Pereira M; Rizzardi K; Mello-da-Silva CA; Impact of exposure to smoke from biomass burning in the Amazon rainforest on human health: A systematic review. J. Bras. Pneumol. 2021, 47, e20210207. 10.36416/1806-3756/e20210207. PMC9013529 34669837

[53] Fernández-Llamazares Á; Garteizgogeascoa M; Basu N; A state-of-the-art review of Indigenous Peoples and environmental pollution. Integr. Environ. Assess. Manag. 2020, 16, 324–341. 10.1002/ieam.4239. PMC7187223 31863549

[54] Butt EW; Conibear L; Knote C; Large air quality and public health impacts due to Amazonian deforestation fires in South America. GeoHealth 2021, 5, e2021GH000429. 10.1029/2021GH000429. PMC8311915 34337273

[55] Hofer CB; Périssé ARS; Vasconcellos ACS; Munduruku Indigenous children: health situational in an area with high mercury exposure. Rev. Saude Publica 2025, 59, e19. 10.11606/s1518-8787.2025059006403 PMC12211787 40608611

[56] Basu N; Horvat M; Zastenskaya I; J. Our evolved understanding of the human health risks of mercury. Ambio 2023, 52, 877–896. 10.1007/s13280-023-01831-6. PMC10073381 36790578

[57] Olivero-Verbel J; Alvarez-Ortega N; Alcala-Orozco M; Population exposure to lead and mercury in Latin America. Curr. Opin. Toxicol. 2021, 27, 27–37. 10.1016/j.cotox.2021.06.002.

[58] Inter-American Commission on Human Rights. Situation of Human Rights of the Indigenous and Tribal Peoples of the Pan-Amazon Region; Inter-American Commission on Human Rights: Washington, DC, USA, 2019.

[59] Inter-American Commission on Human Rights. Indigenous and Tribal Peoples’ Rights over Their Ancestral Lands and Natural Resources; Inter-American Commission on Human Rights: Washington, DC, USA, 2009.

[60] Office of the United Nations High Commissioner for Human Rights. Access to Remedy in Cases of Business-Related Human Rights Abuse: An Interpretive Guide; Office of the United Nations High Commissioner for Human Rights: Geneva, Switzerland, 2024.

[61] Vieira A; Moret A. Caminhões a Diesel na Amazônia: Análise Dos Impactos e Alternativas Sustentávei. Available online: https://redegta.org/wp-content/uploads/2025/12/Caminhoes-a-Diesel-na-Amazonia_Rede-GTA-.pdf (accessed on 1 July 2026).

[62] CHI^2^. Child Heat Impacts & Climate Health Interventions. Available online: https://chi2.ukzn.ac.za/ (accessed on 1 July 2026).

[63] Moges DM; Mattisson K; Malmqvist E; Remote sensing of urban heat dynamics and the cooling effect of urban green spaces in Ethiopian cities. Environ. Chall. 2026, 23, 101462. 10.1016/j.envc.2026.101462.

[64] ENABLE. Enabling Environments for NCD Risk Reduction in Ethiopia. Available online: https://www.gacd.org/research/projects/hc11 (accessed on 1 July 2026).

[65] Koch S; Buekers J; Espinosa A; Association between objectively assessed physical activity and kidney function among female agricultural workers in hot environments in Spain. Environ. Res. 2025, 276, 121420. 10.1016/j.envres.2025.121420. 40113058

[66] INTERCAMBIO. Construction Workers. INTERCAMBIO EU Project. Available online: https://intercambio-project.eu/construction-workers/ (accessed on 1 July 2026).

[67] Gondhiya N; Rehman AU; Andreescu D; Portable electrochemical sensors for per- and polyfluoroalkyl substances: Design, challenges, and opportunities for field deployment. Curr. Opin. Electrochem. 2025, 53, 101725. 10.1016/j.coelec.2025.101725.

[68] Hassan MH; Khan R; Andreescu S. Advances in electrochemical detection methods for measuring contaminants of emerging concerns. Electrochem. Sci. Adv. 2022, 2, e2100184. 10.1002/elsa.202100184.

