# Supplementary material for: Climate, Toxic Exposures and Work: Linking the Amazon, Africa, and Southern Europe through a COP30 Dialogue
Source: Work Health. Author manuscript; Available in PMC 2026 Oct 10. (PMC13652508; doi:10.53941/wah.2026.100017)
Supplement: Supplementary file 1 [file NIHMS2201774-supplement-Supplementary_Material.pdf]

POLICY BRIEF – COP30

Amazon & Heat: Community-First Climate–Environment–Health Action

From the Amazon Basin to Africa and Europe

Cross-Cutting Challenges

- **Combined Exposure, Not Single Hazards**
  Populations are exposed to multiple factors, including heat and chemicals, alongside psychosocial stress and inequalities. Current regulations and risk assessments rarely address these exposure scenarios.
- Disparities, Access to Care and Legal Remedies
  Indigenous populations, workers in precarious jobs and low-income communities carry the heaviest burden but have limited access to basic preventive measures and primary health care infrastructure, and limited access to courts and appropriate compensation.
- **Data Gaps and Fragmented Evidence**
  - Still limited intervention studies on occupational heat in LMICs and pregnant women.
  - Limited longitudinal cohort data capturing early-life and long-term exposures, and health impacts.
  - Lack of standardized indicators for heat-related child health and community-level evaluation.
- **Technology Without Local Ownership**
  Wearables, remote sensing, and omics can support "precision public health", if embedded in locally owned systems, co-designed with communities and linked to policy change.

Priority Policy Actions

1. **Formalize an Amazon Climate–Environment–Health Consortium**
   - **a)** Support a multi-country, multi-institution consortium that:
     - **i)** Links existing and new human cohorts across the Amazon.
     - **ii)** Harmonizes 'environmental and bio-monitoring' (including mercury, microplastics, agrochemicals, air pollution, heat), and health impacts.
     - **iii)** Integrates public health, epidemiology, social science, Indigenous knowledge and legal expertise.
     - **iv)** Ensure Indigenous organizations and local communities share governance and agenda-setting.
     - v) Improve local infrastructure and support the retention of qualified human resources in the Amazon

2) **Create Infrastructure and Administrative Compensation Mechanisms for Indigenous Peoples and traditional communities**
   - **a)** Commit to minimum infrastructure standards in affected territories: safe drinking water, fresh food access, affordable and safe transport, and affordable primary health care.
   - **b)** Establish administrative compensation schemes for Indigenous and riverine communities affected by mining, fossil fuel extraction, logistics and industrial activities.
   - **c)** Expand the application of cost-internalization mechanisms where responsible parties finance remediation and health and environmental services.

3) **Strengthen Environmental Contamination Control in Gold Mining and Oil Extraction**
   - **a)** Rigorously implement and enforce the commitments of the Minamata Convention and other international agreements, including:
     - **i)** The reduction and progressive elimination of mercury use in gold mining;
     - **ii)** Strict control and elimination of other chemicals, including microplastics, agrochemicals, and diesel exposure in workplaces and communities.
   - **b)** Require robust and participatory occupational and environmental and health impact assessments for existing mining and oil extraction projects, including:
     - **i)** Plans for continuous monitoring of contamination in water, soil, air, and food, using novel biosensing approaches;
     - **ii)** Biomonitoring programs in exposed populations (pregnant women, children, and workers).
   - **c)** Ensure health surveillance and clinical follow-up for exposed communities and workers with:
     - **i)** Standardized protocols for biomonitoring, diagnosis, treatment, and rehabilitation of clinical effects;

    - **ii)** Toxicology reference centers accessible to Amazonian populations.
    - **iii)** Implement specific National Policies to prevent human exposure.
  - **d)** Create or strengthen accountability mechanisms for mining and oil companies, including:
    - **i)** Clear obligations for environmental remediation and health compensation;
    - **ii)** Data transparency on emissions, spills, waste discharges, and the use of hazardous substances.
  - **e)** Support clean technologies, fossil fuel phase out, and alternative economic pathways (e.g., mercury-free production chains, occupational transition strategies that account for social and economic impacts on workers for artisanal miners) as an integral part of climate mitigation and adaptation strategies.

4) **Move to Climate-Compatible, Just Logistics in the Amazon**
  - **a)** Set time-bound targets to replace diesel truck fleets with zero-emission vehicles, combined with revitalized low-carbon river transport and restrict new roads.
  - **b)** Condition fiscal and trade incentives (e.g. SUFRAMA[*]) on:
    - **i)** Transparent Scope-3[∞] emissions reporting;
    - **ii)** Progressive decarbonization of logistics chains;
    - **iii)** Full free, prior and informed consent (FPIC) for infrastructure projects.
  - **c)** Install community-accessible air-quality and heat sensors along logistics corridors and make data publicly available.

5) **Protect Workers and Children from Heat**
  - **a)** Update occupational health standards (agriculture, construction, mining, transport) to account for climate-driven heat, including:
    - **i)** Mandatory rest–work–hydration regimes;
    - **ii)** Heat-safe work scheduling;
    - **iii)** Protections for pregnant workers.
  - **b)** Scale up child-focused heat interventions (as in CHI[2]): school infrastructure improvements, shaded play areas, early warning, and education.

6) **Build Shared Indicators, Data Systems and Capacity**
  - **a)** Develop a common indicator set for heat- and pollution-related health impacts.
  - **b)** Fund cross-regional training programs (Amazon–Africa–Europe) for early-career researchers, health workers, and community leaders, improve local infrastructure for data analysis, and support long-term fixation of qualified human resources.
  - **c)** Support open, interoperable data platforms that respect community data sovereignty and enable joint analyses of exposure mixtures and health outcomes.

Call to Action

Protecting Amazonian people and forests, African urban communities, and workers on all continents from the combined risks of heat and pollution is both a public health imperative and a fundamental condition for climate mitigation and adaptation strategies that reduce disproportionate health and environmental impacts. This according to the goal of ensuring clean, healthy, and sustainable environments, consistent with resolutions by the UN Human Rights Council and General Assembly, which frame these issues as central to internationally recognized health and environmental standards.

---

[*] **SUFRAMA** (Superintendência da Zona Franca de Manaus), is the Brazilian federal agency that manages the Manaus Free Trade Zone and other special economic areas in the Amazon region. Its primary role is to administer tax incentives and fiscal benefits to businesses in these areas to promote regional development, such as exemptions on various taxes

[∞] **Scope 3** refers to all indirect greenhouse gas emissions that occur in a company's value chain, beyond those directly owned or controlled by the company (Scope 1) and those from purchased energy (Scope 2).
